# Fall armyworm migration across the Lesser Antilles and the potential for genetic exchanges between North and South American populations

**DOI:** 10.1371/journal.pone.0171743

**Published:** 2017-02-06

**Authors:** Rodney N. Nagoshi, Shelby Fleischer, Robert L. Meagher, Mirian Hay-Roe, Ayub Khan, M. Gabriela Murúa, Pierre Silvie, Clorinda Vergara, John Westbrook

**Affiliations:** 1 Center for Medical, Agricultural and Veterinary Entomology, USDA-ARS, Gainesville, Florida, United States of America; 2 Department of Entomology, The Pennsylvania State University, University Park, Pennsylvania, United States of America; 3 Department of Life Sciences, The University of the West Indies, Saint Augustine, Trinidad & Tobago; 4 Instituto de Tecnología Agroindustrial de Noroeste Argentino (ITANOA), Consejo Nacional de Investigaciones Científicas y Técnicas (CONICET), Estación Experimental Agroindustrial Obispo Colombres (EEAOC), Tucumán, Argentina; 5 CIRAD, UPR Agroécologie et Intensification Durable des cultures Annuelles, F-34398 Montpellier, France; 6 IRD, UR 072, Laboratoire Evolution, Génomes et Spéciation, Orsay, France; 7 Museo de Entomologia Klaus Raven Buller, Universidad Agraria La Molina, Lima, Peru; 8 Insect Control and Cotton Disease Research Unit, USDA-ARS, College Station, Texas, United States of America; National Cheng Kung University, TAIWAN

## Abstract

The fall armyworm, *Spodoptera frugiperda* (J. E. Smith)(Lepidoptera: Noctuidae), is an important agricultural pest of the Western Hemisphere noted for its broad host range, long distance flight capabilities, and a propensity to develop resistance to pesticides that includes a subset of those used in genetically modified corn varieties. These characteristics exacerbate the threat fall armyworm poses to agriculture, with the potential that a resistance trait arising in one geographical location could rapidly disseminate throughout the hemisphere. A region of particular concern is the Caribbean, where a line of islands that extends from Florida to Venezuela provides a potential migratory pathway between populations from North and South America that could allow for consistent and substantial genetic interactions. In this study, surveys of populations from Peru, Bolivia, Paraguay, and Trinidad & Tobago expand on previous work in South America that indicates a generally homogeneous population with respect to haplotype markers. This population differs from that found in most of the Lesser Antilles where a combination of genetic and meteorological observations is described that indicate fall armyworm migration from Puerto Rico to as far south as Barbados, but does not support significant incursion into Trinidad & Tobago and South America. Air transport projections demonstrate that the wind patterns in the Caribbean region are not conducive to consistent flight along the north-south orientation of the Lesser Antilles, supporting the conclusion that such migration is minor and sporadic, providing few opportunities for genetic exchanges. The implications of these findings on the dissemination of deleterious traits between the two Western Hemisphere continents are discussed.

## Introduction

Adventive (non-native) insect pests are a major concern for agriculture in all countries. The highest percentage of non-indigenous insects in the continental United States is found in Florida, with the Caribbean region the most likely source of natural arthropod migration into Florida by flight [1, 2]. A related issue is the spread of deleterious traits, such as pesticide resistance, which could disseminate rapidly within even a widely distributed species if there are opportunities for substantial genetic interactions between geographical populations. Essential for assessing the risk of such events is an understanding of the migratory behavior of the pest in question. An advantageous Lepidoptera model for studying migratory processes, particularly as it relates to pest infestation patterns, is the noctuid moth *Spodoptera frugiperda* (J. E. Smith) (Lepidoptera: Noctuidae), commonly known as the fall armyworm. It is an important pest of corn (*Zea mays* L.), sorghum (*Sorghum vulgare* Pers.), cotton (*Gossypium hirsutum* L.), and several turf grass species [3] in both North and South America.

The capacity for long-distance migration was demonstrated for fall armyworm populations in North America, where infestations occur throughout the continental U.S. and parts of southern Canada despite the intolerance of the species to the prolonged winter freezes that affect most of this range [4–6]. This infestation pattern is explained by overwintering in southern Texas and Florida, followed by annual re-invasions of its northern geographic range through successive long-distance flights during the spring and summer. The direction and extent of these movements are strongly influenced by meteorological factors, most notably wind systems [7]. Several studies have investigated how synoptic wind patterns affect the frequency, intensity, and displacement of migratory flights of noctuid moths [8–10]. It was demonstrated that weather transport systems are the most important climatic factors governing fall armyworm abundance at a migratory destination [11], that the direction of migratory flight is highly correlated with wind headings [12], and that migratory pathways can be modeled using projections of air transport trajectories [13].

Amplified fragment length polymorphism (AFLP) analysis of fall armyworm populations from widely scattered locations in the Western Hemisphere describes a genetically diverse species that exists as a single interbreeding population [14]. However, other genetic data indicate a more complex picture. Mitochondrial haplotype comparisons identified two subgroups that displayed reproducible biases in host plant distribution. These groups were designated "strains" and named for the host plant from which they were originally collected [15]. The rice-strain was subsequently shown to have a variable attraction to rice, but is consistently associated with pasture and turf grass species as well as alfalfa [16, 17]. The corn-strain predominates in corn habitats and is also associated with cotton as a secondary host [18–20]. The two strains are sympatric so mating barriers of some type must exist, which may involve directional hybrid sterility [21].

Geographical genetic structure was demonstrated for the corn-strain subpopulation using a haplotype methodology that had limited resolution but was less sensitive to the homogenizing effects of gene flow than AFLP. The corn-strain can be subdivided into four haplotype categories (CSh1-4) based on polymorphisms in the mitochondrial *CO1* gene [22]. Overwintering populations in Texas and Florida differ in the proportions of two haplotypes (CSh2 and CSh4), making possible a genetic mapping of corn-strain migration patterns in North America [23–25]. Substantial overlaps in the migration from the Texas and Florida overwintering locations make genetic interactions between the two populations likely [26], but these have not been sufficient to homogenize the haplotype ratios based on over a decade of monitoring [27]. Subsequent studies demonstrated that the Florida haplotype profile (designated FAW[FL]) extends to Puerto Rico fall armyworm, while populations in Brazil and Argentina are uniformly of the Texas profile, or FAW[TX] [6, 24, 28]. Equivalent markers have not been found for the rice-strain so a similar analysis of this group is not currently possible.

**Fig 1:**
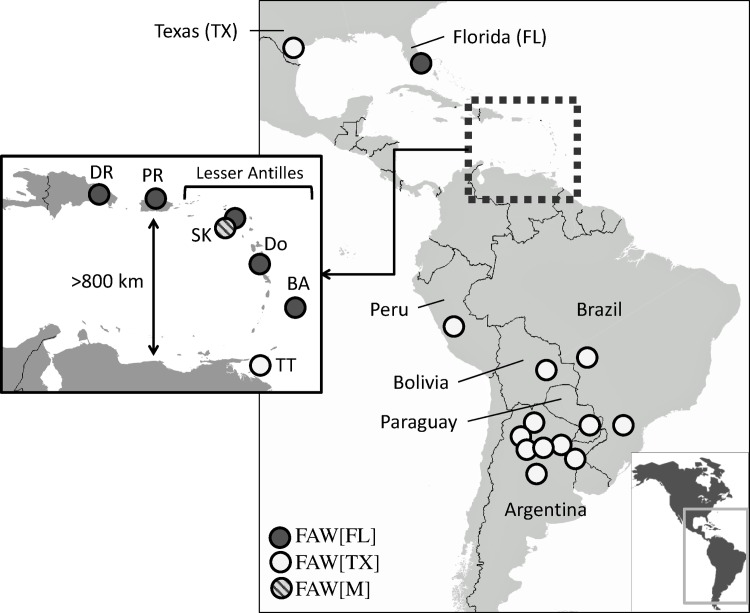
Map of fall armyworm haplotype ratios in the Caribbean and South America. Circles approximate locations of samplings and display the observed haplotype ratio category. If multiple collections from the same location have the same haplotype ratio type a single circle is presented. Ratios for Argentina and Brazil are a summation of data from previous studies [6, 17, 28] (S1 Table). FL, Florida; DR, Dominican Republic; PR, Puerto Rico; SK, St. Kitts and Nevis; Do, Dominica; Ba, Barbados; TT, Trinidad & Tobago.

The observed haplotype difference between the corn-strain populations of the two Western Hemisphere continents make it possible to examine the degree to which these two groups might interact. One area where this might occur is the southern Caribbean region between Puerto Rico and South America. These two landmasses are separated by over 800 km of water, a distance unlikely to be traversed by a moth in a single flight (Fig 1). However, a plausible migratory pathway could involve short hops across the Lesser Antilles, a line of small islands to the east that includes Trinidad & Tobago to the south and extends to, but does not include, Puerto Rico to the northwest.

Because both fall armyworm strains are polyphagous with a broad host range that include over 80 plant species [3, 19], the availability of host plants is probably not a limiting factor for migration in this region. More restrictive will be the small sizes of the islands. The Lesser Antilles has a total land area of about 15,100 km^2^, of which about a third belongs to Trinidad & Tobago. The remaining islands range in area from 13 km^2^ to 1800 km^2^, sizes that can only be expected to support a small local population that are prone to abrupt changes in genetic composition or numbers due to genetic drift or migration (reviewed in [29]). In contrast, the more expansive island of Puerto Rico (9,104 km^2^) supports large and stable fall armyworm populations [24, 30, 31]. Given these factors, our premise is that fall armyworm populations in Puerto Rico to the north and South America to the south were the principle sources for island-scale immigration in the intervening islands of the Lesser Antilles.

Because these putative source populations have different haplotype profiles, it should be possible to extrapolate the origins of the island fall armyworm by haplotype analysis and, just as importantly, estimate the extent to which the two source populations mix and interbreed in the Caribbean region. Specifically, there are three haplotype patterns anticipated. Islands sourced primarily from Puerto Rico would exhibit a majority CSh4 haplotype, the FAW[FL] profile first described in Florida, while populations resulting from South American migrations would be predominantly CSh2 as observed in Texas (FAW[TX]). Substantial and frequent migration in one or both directions will result in the introgression of one population into the other, generating a mixing of both CSh2 and CSh4 in proportions intermediate to FAW[FL] and FAW[TX]. This "mixed" category (designated FAW[M]) is consistently observed in the southeastern and northeastern portions of the United States where the Florida and Texas migratory pathways are thought to overlap [26].

In this study, wind vector data for the southern Caribbean region were summarized for different time periods and compared to haplotype frequencies in select populations. Additional comparisons were made with trajectory simulations from different locations in the Caribbean using an air trajectory model. These observations were used to extrapolate the likelihood and magnitude of genetic exchanges in the Lesser Antilles between the populations from the two Western Hemisphere continents. The implications of these results on the spread of a pesticide resistance trait are discussed.

## Materials and methods

### Specimen collections and sites

Specimens were obtained either as larvae collected directly from corn host plants or as adult males from pheromone traps located in or near cornfields. In the larval collections from 2014–2015 in the Caribbean region, efforts were made to reduce the possibility of collecting multiple specimens from the same egg mass. This involved selecting only a single larva from an infested plant and none from adjacent plants. This method was not used in the earlier 2013 larval collections from Trinidad & Tobago, which were from two locations within the town of Mount Hope and designated TT-A and TT-B. However, polymorphisms in the *CO1* region indicated that TT-A and TT-B were derived from at least nine and three different maternal lineages, respectively. All larval specimens were killed by freezing and stored either air-dried or in ethanol at room temperature.

Pheromone trap collections in the Caribbean used white plastic delta traps (AlphaScents, Inc., West Linn, OR) with No-mess sticky card^TM^ inserts baited with one of two versions of the sex pheromone blend (four-component, L105A, and two-component, L976) (Scentry Biologicals, Inc., Billings, MT). Both lures were shown to be equally effective in attracting males of both strains [32]. The delta traps were hung from 1.5m poles constructed from hard alloy aluminum tubing (2cm) net handles (BioQuip Products, Rancho Dominguez, CA; 7300 Professional Series). Pheromone collections in Florida were performed using standard (green top, yellow funnel, white bucket) or all-green Universal moth traps (Unitraps) (Great Lakes IPM, Vestaburg, MI). Each trap contained insecticide strips containing 10% 2,2-dichlorovinyl dimethyl phosphate (Hercon® Environmental, Emigsville, PA) to kill moths. Specimens were identified as fall armyworm by morphological criteria before molecular analysis.

The location and designation of each collection is listed below and coordinates are provided in Table 1. An initial 2013 larval collection from Christ Church parish in Barbados (Ba-A) provided only a few (12) specimens. Additional Barbados collections were done in 2015 in the parishes of Kendall (Ba-B), Saint George (Ba-C), Saint Philip (Ba-D), and Saint James (Ba-E). Specimens from Dominica were from Saint Peter parish (Do-A, Do-B). St. Kitts and Nevis collections were from the parishes of Saint Anne Sandy Point (SK-A, SK-B, SK-C), Saint George Basseterre (SK-D), Saint Paul Capesterre (SK-E), and Christ Church Nichola Town (SK-F). Dominican Republic collections were made in Santo Domingo province (DR-A, DR-B). Paraguay collections were made near the cities of Caacupé (Par-A, Par-C) and Guayaibi (Par-B). The Bolivia collection was near the city of Cotoca (Bol). The collection in Peru were made in the district of La Molina (Per-A, Per-B). The Florida collection (FL-A) include specimens pooled from pheromone trapping done in the counties of Alachua, Hendry, Hillsborough, Martin, Miami-Dade, Orange, Palm Beach, and Suwanee. Additional data are from specimens described previously (Table 1).

**Table 1:** Source information for fall armyworm collections.

Name	Location	Date	Type^a^	Coordinates/Reference
TT-A	Trinidad & Tobago	2013	L	10°39'16”N 061°26'03"W
TT-B	Trinidad & Tobago	2013	L	10°39'16”N 061°26'03"W
Ba-A	Barbados	2013	L	13°04'22”N 059°31'33"W
Ba-B	Barbados	2015	L	13°08'42”N 059°29'57"W
Ba-C	Barbados	2015	T	13°08'24”N 059°32'45"W
Ba-D	Barbados	2015	T	13°07'32”N 059°27'21"W
Ba-E	Barbados	2015	T	13°11'03”N 059°37'49"W
Do-A	Dominica	2014	L	15°30'35”N 061°26'57"W
Do-B	Dominica	2014	T	15°30'35”N 061°26'57"W
SK-A	St. Kitts and Nevis	2014	L	17°22'21”N 062°50'38"W
SK-B	St. Kitts and Nevis	2015	L	17°22'21”N 062°50'38"W
SK-C	St. Kitts and Nevis	2015	T	17°22'21”N 062°50'38"W
SK-D	St. Kitts and Nevis	2015	T	17°14'06”N 062°38'39"W
SK-E	St. Kitts and Nevis	2015	T	17°24'06”N 062°49'32"W
SK-F	St. Kitts and Nevis	2015	T	17°21'37”N 062°45'42"W
DR-A	Dominican Republic	2015	T	18°29'09”N 069°55'52"W
DR-B	Dominican Republic	2016	T	18°29'09”N 069°55'52"W
Par-A	Paraguay	2005	L	25°22'51”S 057°09'27"W
Par-B	Paraguay	2005	L	24°31'33”S 056°24'27"W
Par-C	Paraguay	2006	L	25°22'51”S 057°09'27"W
Bol	Bolivia	2012	L	17°46'21”S 062°52'24"W
Per-A	Peru	Jun 2014	T	12°04'55”S 076°55'41"W
Per-B	Peru	Oct 2014	T	12°04'55”S 076°55'41"W
FL-A	Alachua County	2012–5	T	29°39'28”N 082°18'06"W
FL-A	Hendry County	2012–5	T	26°36'37”N 081°04'31"W
FL-A	Miami-Dade County	2012–5	T	25°33'05”N 080°37'57"W
FL-A	Orange County	2012–5	T	28°29'04”N 081°15'06"W
FL-A	Palm Beach County	2012–5	T	26°39'05”N 080°16'36"W
FL-A	Suwanee County	2012–5	T	30°14'54”N 082°59'35"W
Published data				
FL-B	Florida	2004–11	T+L	[25]
PR-A	Puerto Rico	2007	T	[33]
PR-B	Puerto Rico	2009	T	[33]
Brz	Brazil	2005–7	L	[28]
Arg-A	Argentina	2010–11	C	[34]
Arg-B	Argentina	2012	L	[35]

^a^ L, Larvae collection; T, Pheromone trapping; C, Laboratory colonies.

### DNA preparation

Individual specimens were homogenized in 1.5 ml of phosphate buffered saline (PBS, 20 mM sodium phosphate, 150 mM NaCl, pH 8.0) using a tissue homogenizer (PRO Scientific Inc., Oxford, CT, USA) and the homogenate transferred to a 2-ml microcentrifuge tube. Cells and tissue were pelleted by centrifugation at 6000 g for 5 min. at room temperature. The pellet was resuspended in 800 μl Genomic Lysis buffer (Zymo Research, Orange, CA, USA) by vortexing and incubated at 55°C for 5 min. Debris was removed by centrifugation at 10,000 rpm for 3 min. The supernatant was transferred to a Zymo-Spin III column (Zymo Research, Orange, CA, USA) and processed according to manufacturer’s instructions. The DNA preparation was increased to a final volume of 100 μl with distilled water. Genomic DNA preparations of fall armyworm samples from previous studies were stored at -20°C.

### Characterization of the *CO1* haplotypes

PCR amplification of the mitochondrial *CO1* gene was performed in a 30-μl reaction mix containing 3 μl 10X manufacturer’s reaction buffer, 1 μl 10mM dNTP, 0.5 μl 20-μM primer mix, 1 μl DNA template (between 0.05–0.5 μg), 0.5 unit Taq DNA polymerase (New England Biolabs, Beverly, MA, USA). The thermocycling program was 94°C (1 min), followed by 33 cycles of 92°C (30 s), 56°C (45 s), 72°C (45 s), and a final segment of 72°C for 3 min. Typically 96 PCR amplifications were performed at the same time using either 0.2-ml tube strips or 96 well microtiter plates. Primers were synthesized by Integrated DNA Technologies (Coralville, IA, USA). Amplification of the *CO1* region used the primer pair *CO1-893F* (5’-CACGAGCATATTTTACATCWGCA-3’) and *CO1-1472R* (5’-GCTGGTGGTAAATTTTGATATC-3’) to produce a 603-bp fragment.

For fragment isolations 6 μl of 6X gel loading buffer was added to each amplification reaction and the entire sample run on a 1.8% agarose horizontal gel containing GelRed (Biotium, Hayward, CA, USA) in 0.5X Tris-borate buffer (TBE, 45 mM Tris base, 45 mM boric acid, 1 mM EDTA pH 8.0). Fragments were visualized on a long-wave UV light box and cut out from the gel. Fragment isolation was performed using Zymo-Spin I columns (Zymo Research, Orange, CA, USA) according to manufacturer’s instructions. The isolated fragments were analyzed by DNA sequencing using primers *CO1-893F* or *CO1-1472R*, and was performed by the University of Florida ICBR center. Strain identity was determined by the presence of specific nucleotides at strain-specific polymorphic sites as described in the text. DNA comparisons, alignments, and restriction site mapping were performed using the DS Gene program (Accelrys, San Diego, CA, USA) and the CLUSTAL algorithm. Statistical analyses were performed using GraphPad Prism version 7.00 for Mac (GraphPad Software, La Jolla, CA, USA).

### Calculation of haplotype ratio

DNA sequence analysis of the *CO1* segment identifies four haplotype categories defined by polymorphisms at two loci that together encompass the corn-strain group [22]. Haplotypes CSh1 and CSh3 are generally infrequent, while CSh2 and CSh4 vary by region, with CSh2 predominant in Texas and CSh4 the majority haplotype in Florida. The haplotype profile was originally compared using the ratio CSh4/CSh2, a simple metric that takes into account the relative amounts of CSh2 and CSh4. Initially, the FAW[TX] population was defined by a CSh4/Csh2 ratio less than or equal to 0.5 and FAW[FL] by a ratio greater than or equal to 1.5 [22]. These thresholds were later modified to ≤0.6 (FAW[TX]) and ≥1.3 (FAW[FL]) based on additional data from North America [25]. The CSh4/CSh2 metric was of limited use in this study because there were multiple instances where the number of CSh2 observed was 0, producing an incalculable ratio. To avoid this problem a modified haplotype metric was used (CSh4 –CSh2)/(CSh4 + CSh2) that still directly and solely compares CSh2 to CSh4, but this time varies from -1 (all CSh2) to +1 (all CSh4). The FAW[TX] and FAW[FL] thresholds using this metric were calculated as follows. A 0.6 CSh4/CSh2 ratio occurs when the frequency of CSh4 is 0.6 that of CSh2. Substituting these values into the modified ratio metric, (0.6X-X)/(0.6X+X), produces an equivalent FAW[TX] upper limit of -0.25. A CSh4/CSh2 value of 1.3 occurs when the CSh4 to CSh2 proportion is 4:3. This converts to a new ratio of (4X-3X)/(4X+3X) = 1/7 = 0.13. Based on these calculations, the adjusted category limits for the modified ratio are FAW[FL] when the ratio is greater than or equal to 0.1 (with a maximum of 1.0 when CSh2 = 0), FAW[TX] when the ratio is less than or equal to -0.3 (with a minimum of -1.0 when CSh4 = 0), and a "mixed" profile called FAW[M] with intermediate values, -0.3 < ratio < 0.1.

### Wind climatology maps

Wind vector maps were generated from monthly average wind velocity (vector mean wind speed and wind direction) using the 1981–2010 base period derived from NCEP-NCAR reanalysis of monthly zonal (i.e., westerly) and meridional (i.e., southerly) wind velocity components at a pressure-height of 925 mb (hPa) (source: National Oceanic and Atmospheric Administration, NOAA, and the National Centers for Environmental Prediction, NCEP). The analyzed maps were obtained online from the International Research Institute for Climate and Society (Earth Institute, Columbia University, New York, NY; http://iridl.ldeo.columbia.edu/maproom/Global/Climatologies/Vector_Winds.html.

### HYSPLIT trajectory maps

Air transport trajectories for various locations were estimated using the Hybrid Single Particle Lagrangian Integrated Trajectory Model at the Air Resources Laboratory (ARL) READY web site run by NOAA (http://ready.arl.noaa.gov/HYSPLIT.php; [36]. Heliothine moths typically migrate during evening hours, with long-distance flight occurring at below 1000 m AGL (meters Above Ground Level) and for a period of 8 to 12 hours [37–40]. Based on these observations, HYSPLIT projections were made for 12-hour flights beginning at 18:00 h (0000 UTC) in the evening at an altitude of 500 m AGL. Daily trajectories were obtained for the months of January, April, July and October 2015 and displayed as a frequency distribution from four origins, Puerto Rico (PR, 18°13'15”N 066°35'24"W), St. Kitts and Nevis (SK, 17°21'28”N 062°46'59"W), Dominica (Do, 15°24'54”N 061°22'16"W), Barbados (13°11'38”N 059°32'36"W), Trinidad & Tobago (TT, 10°41'30”N 061°13'21"W).

## Results

### Variability in strain proportions

Field samplings were either larval collections from corn hosts or pheromone trapping in the vicinity of cornfields. The corn-strain was the majority in 10 out of 11 larval collections, consistent with previous demonstrations of a general strain bias in host use that was not absolute [41–43]. Greater strain variability was found with the pheromone trap collections, where only five of the 12 collections from the Caribbean region had a corn-strain majority (Table 2). We believe this reflects the small size of the corn plots in most of the Caribbean islands, which means that host plants preferred by the rice-strain were within the attractant range of the pheromone traps. There were two examples where larval and pheromone trap collections performed at approximately the same time and place gave very different results, Dominica (Do-A and Do-B) and St. Kitts and Nevis (SK-B and SK-C), demonstrating the sympatric distribution of the two strains and the importance of taking into account the collection method when extrapolating strain composition.

**Table 2:** Strain and haplotype composition of the fall armyworm collections.

Name	Larva[L] Trap[T]	Total	%CS	CSh1	CSh2	CSh3	CSh4	Ratio^a^	Type^b^
TT-A	L	22	100	5	16	0	1	-0.9	TX
TT-B	L	9	100	0	9	0	0	nd	nd
Ba-A	L	12	8	0	0	0	1	nd	nd
Ba-B	L	91	100	0	0	0	91	1.0	FL
Ba-C	T	15	100	0	0	0	15	1.0	FL
Ba-D	T	64	100	0	0	0	64	1.0	FL
Ba-E	T	61	100	0	0	0	61	1.0	FL
Do-A	L	29	100	0	8	0	21	0.4	FL
Do-B	T	14	29	0	1	0	3	nd	nd
SK-A	L	55	69	0	20	0	18	-0.1	M
SK-B	L	82	68	0	5	6	48	0.8	FL
SK-C	T	33	6	0	0	0	1	nd	nd
SK-D	T	41	0	0	0	0	1	nd	nd
SK-E	T	18	0	0	0	0	0	nd	nd
SK-F	T	8	0	0	0	0	0	nd	nd
DR-A	T	87	13	1	2	3	5	0.4	FL
DR-B	T	107	48	1	14	1	35	0.4	FL
Pgy-A	T	4	75	2	4	0	0	nd	nd
Pgy-B	T	9	67	1	2	0	0	nd	nd
Pgy-C	L	18	100	7	10	0	1	-0.8	TX
Bol	L	71	80	8	49	0	0	-1.0	TX
Per-A	L	35	100	9	26	0	0	-1.0	TX
Per-B	L	48	90	13	30	0	0	-1.0	TX
FL-A	T	nd	nd	40	222	8	533	0.4	FL
FL-B	T+L	nd	nd	28	106	4	342	0.5	FL
PR-A	T	nd	nd	10	22	8	67	0.5	FL
PR-B	T	nd	nd	3	20	5	94	0.6	FL
Brz	L	nd	nd	37	130	5	5	-0.9	TX
Arg-A	C	nd	nd	12	89	0	0	-1.0	TX
Arg-B	L	nd	nd	14	68	0	0	-1.0	TX

^a^ Ratio is (CSh4-CSh2)/(CSh4+CSh2), ratio was not done (nd) if corn strain sample size was less than ten.

^b^ FL: FAW[FL] if ratio ≥ 0.1 with a maximum of 1.0 when CSh2 = 0; TX: FAW[TX] if ratio ≤ -0.3 with a minimum of -1.0 when CSh4 = 0; FAW[M] with intermediate values, -0.3 < ratio < 0.1.

### Haplotype profiles in the Lesser Antilles

In contrast to host strain proportions, both larval and pheromone trap collections gave similar corn-strain haplotype profiles, with generally consistent results for each location (Table 2). Populations from both Florida and Puerto Rico were previously shown to have the same haplotype configuration (FAW[FL]) that is distinct from the FAW[TX] profile found in the overwintering sites of Texas or in the South American countries of Argentina and Brazil [6, 28]. Fall armyworm in the Dominican Republic, which lies between Florida and Puerto Rico, were also of the FAW[FL] configuration (Table 2), as were collections from Dominica and Barbados. The Barbados collections represented an extreme case where all 232 specimens had the CSh4 haplotype (Fig 1). At the other extreme were collections from Trinidad & Tobago (TT-A, TT-B), which were of the FAW[TX] profile but in this case with only one of the 31 corn-strain specimens tested of the CSh4 haplotype. The only location where multiple collections produced different haplotype profiles was in St. Kitts and Nevis, where the SK-A collection showed the FAW[M] profile with a near equal number of CSh4 and the CSh2 haplotypes, while SK-B displayed a FAW[FL] configuration.

### Air transport patterns in the Caribbean

**Fig 2:**
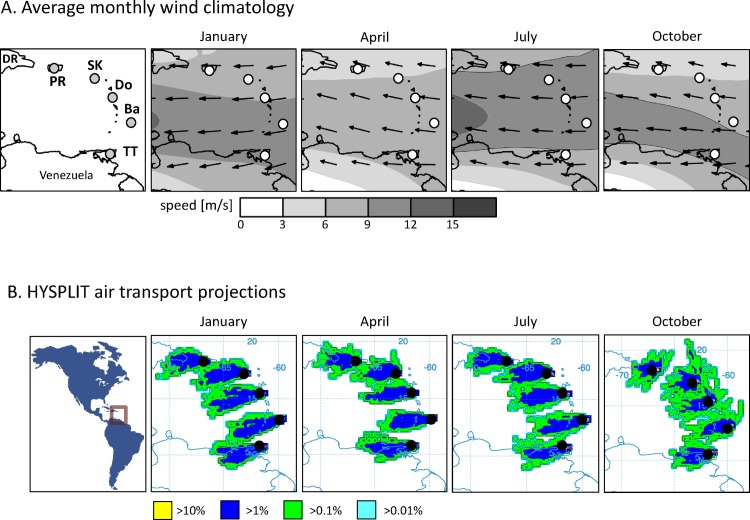
Description of vector mean winds and haplotype profiles in the Caribbean region. A. Climatology of monthly vector mean winds at the 925-mb pressure-height from selected months from NCEP-NCAR Reanalysis data using the 1981–2010 base period. Vector mean wind speed is greyscale-coded and arrows denote vector mean wind direction. Circles indicate the location of specimen collections. B. HYSPLIT forward projections of 12-h overnight trajectories at 500 m AGL based on historical wind data. Average monthly frequency distribution of trajectories for selected months are displayed from Puerto Rico (PR), St. Kitts and Nevis (SK), Dominica (Do), Barbados (Ba), Trinidad & Tobago (TT). Percentage of projected trajectories is indicated by color.

The dominant influence on wind patterns in the Caribbean region is the Caribbean Low Level Jet, an easterly zonal wind system with a maximum speed at a pressure-height of 925 mb that corresponds to an altitude of approximately 600–800 m AGL [44]. The result is a predominantly easterly wind flow over Puerto Rico and the islands of the Lesser Antilles throughout the year (Fig 2A).

The HYSPLIT atmospheric trajectory model was used to simulate nightly wind-directed dispersal of fall armyworm from the sampling sites (Fig 2B). Projections were made at 500 m AGL, a height previously shown to be associated with noctuid moth migration in Texas and within the altitude range where flying specimens could be captured using nets towed by fixed-wing aircraft [12]. HYSPLIT projections made at 850 m AGL and 1000 m AGL gave similar patterns (data not shown). Four sets of projections were calculated representing the monthly average frequency of single-night trajectories for January, April, July, and October using climate data from 2014. These months represent periods when wind speeds show substantial seasonal differences, with a pattern of highest speed in July, a minimum observed in October, a secondary peak in January, and a minor decline in April-May [45]. The results were generally consistent with what would be expected from the predominant easterly wind flow. Most of the projected trajectories terminated over water, with a few indications that regular transport between the islands was possible (Fig 2B). Trajectories from Puerto Rico had substantial overlap with the Dominican Republic, a distance of less than 200 km. A broad frequency distribution pattern was observed emanating from St. Kitts and Nevis that overlapped the eastern coast of Puerto Rico and smaller neighboring islands. Similarly, trajectories from Barbados passed over several nearby islands lying to the west, while trajectories calculated from Trinidad & Tobago tended westward into the South American continent. The broadest frequency distribution patterns were found in October, a period typically associated with reduced seasonal wind speeds.

### Prevailing winds and haplotype patterns in South America

**Fig 3:**
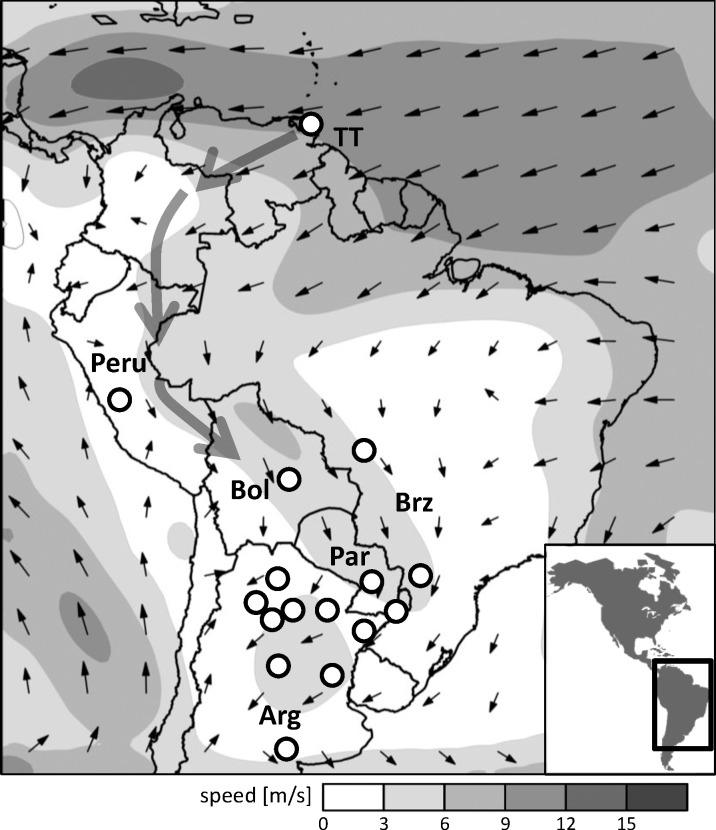
Description of vector mean winds and haplotype profiles in South America. Climatology of monthly vector mean winds from January at the 925-mb pressure-height from NCEP-NCAR Reanalysis data using the 1981–2010 base period superimposed on map of haplotype ratio sampling. Large arrows indicate expected population movement trajectories from Trinidad & Tobago based on the prevailing wind patterns. TT, Trinidad & Tobago; Bol, Bolivia; Par, Paraguay; Arg, Argentina; Brz, Brazil.

The South American Low Level Jet is the predominant lower altitude wind system in South America. It initiates as an easterly trade wind from the equatorial Atlantic that is deflected sharply southward by the higher elevations of the Andes Mountain Range [46]. The south-southeastward deflection occurs just north of and passes through Bolivia, with highest wind speeds occurring during the austral spring and summer seasons (Fig 3). Fall armyworms in Bolivia are therefore likely to regularly contribute to populations to the southern and eastern portions of the continent. The haplotype data are consistent with this scenario. Previous studies indicated that fall armyworm populations from Brazil and Argentina were dominated by the CSh2 haplotype [6, 28, 42]. We found the same to be true for fall armyworm collections from Paraguay, Bolivia, and Peru. Of the 162 corn-strain specimens tested from these three countries, only one was CSh4 (Table 2). This is consistent with a generally homogeneous haplotype configuration for populations in South America (Fig 3).

## Discussion

### Limited genetic exchange between Florida and South American fall armyworm populations

**Fig 4:**
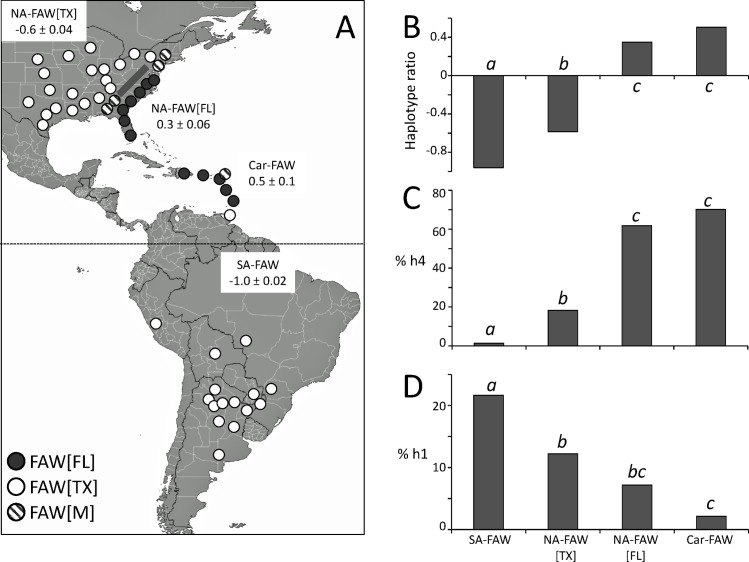
Fall armyworm haplotype ratio distributions in the Western Hemisphere. A. Hemispheric map of haplotype ratio categories and their segregation into groups based on geography and haplotype ratios. NA-FAW[TX] and NA-FAW[FL] represent collections from the United States with a FAW[TX] or FAW[FL] haplotype ratio profile, respectively. Car-FAW includes collections from the Caribbean not including Trinidad & Tobago. SA-FAW includes collections from South America and Trinidad & Tobago. The mean ratio ± the Standard Error of the Mean (SEM) is indicated. Diagonal line indicates location of the Appalachian mountain range. United States data are from an earlier study [25]. South America data are as described in Fig 1. Graphs B, C, D describe ordinary one-way ANOVA analysis with Tukey's multiple comparisons test. Columns with different lower case letters are significantly different at the *P* < 0.05 criterium. B. Comparisons of haplotype ratios (*F(3*, *31)* = 113.2, *P* < 0.0001, *r*^*2*^ = 0.9164). C. Comparisons of the h4 haplotype frequency (*F(3*, *31)* = 99.58, *P* < 0.0001, *r*^*2*^ = 0.9060). D. Comparisons of the h1 haplotype frequency (*F(3*, *31)* = 15.73, *P* < 0.0001, *r*^*2*^ = 0.6035). Data used in the ANOVA analysis are in S1 Table.

In a previous study we demonstrated that in North America, the boundary between FAW[FL] and FAW[TX] coincides with the Appalachian mountain range, which may be contributing to the separation of the two migratory waves originating from Florida and Texas (Fig 4A, [25]). The FAW[M] profile is consistently observed at the southern and northern ends of the Appalachian mountain range, indicating possible hybrid zones where there is mixing of the FAW[TX] and FAW[FL] groups (arrows, Fig 4A). This is of genetic consequence as it provides an opportunity for genetic exchange between the two overwintering populations that are otherwise geographically segregated. The rarity of the FAW[M] profile in the Caribbean suggests limited opportunities for genetic exchanges in this region between North American and South American fall armyworm populations. Supporting evidence for this comes from comparisons of four groups defined by geography and haplotypes. These are NA-FAW[TX] and NA-FAW[FL] representing North American populations expressing either the FAW[TX] or FAW[FL] haplotype profile, respectively, the Caribbean fall armyworm (not including Trinidad & Tobago) denoted as Car-FAW, and the samplings from South America and Trinidad & Tobago (SA-FAW). The mean haplotype ratio of the NA-FAW[TX] collections is significantly different from the SA-FAW group (Fig 4B), which corresponds to a statistically significant difference in the proportion of the CSh4 haplotype between these groups, with a NA-FAW[TX] mean of 18 ± 2% compared to only 2 ± 1% for SA-FAW (Fig 4C). The results are consistent with the assumption that the higher incidence of the FAW[M] profile in the United States provides increased opportunities for the introgression of the CSh4 haplotype into the Texas overwintering population, with this haplotype now present in North America at levels that are generally intermediate to those spanned by the Caribbean and South American populations.

Comparable indications were found with the distributions of the CSh1 haplotype, typically a minor component of the corn-strain population (Fig 4D). This haplotype is found in 23% of the SA-FAW specimens, but in only 1% of the pooled collections from Car-FAW. The North American populations are again at intermediate levels, with the NA-FAW[TX] CSh1 frequency significantly different from both the Caribbean and South American populations. The NA-FAW[FL] CSh1 frequency is similar to that of the NA-FAW[TX] group, lower than that observed in South America and higher than that measured in the Caribbean, though the latter was not statistically significant. As with CSh4, the CSh1 distribution pattern is consistent with there being sufficient genetic interactions between the North American overwintering populations to cause a change in the haplotype profile, with the trend being to homogenize the haplotype differences. In contrast, there is no evidence for comparable introgressions between Caribbean and South American populations.

### Fall armyworm movements in the Lesser Antilles

Fall armyworm from the Dominican Republic in 2015–2016 exhibited the same haplotype profile as those surveyed in neighboring Puerto Rico in 2007 and 2009, consistent with a permanent population extending northward to Florida that is characterized by CSh4 as the majority haplotype and CSh2 present as a secondary but substantial (15%-37%) component (Table 2). Similar haplotype results were obtained for 2014 collections from St. Kitts and Nevis and Dominica, but a more extreme phenotype was obtained for collections from Barbados where all 231 specimens from four locations surveyed in 2015 were of the CSh4 haplotype. Given the near absence of CSh4 in the collections from Trinidad & Tobago the source of the CSh4 haplotype in the Lesser Antilles is most likely Puerto Rico, indicating that movement of the FAW[FL] population to at least Barbados does occur (Fig 4A).

These findings also argue against South American fall armyworm undergoing significant northward migration beyond Trinidad & Tobago. The only evidence supporting such an introgression occurred in St. Kitts and Nevis in 2014 (St. Kitts-A), where a FAW[M] profile with a near equal number of CSh2 and CSh4 was observed (Table 2). However, this collection differs from that of the nearby islands of Puerto Rico and Dominica as well as that observed in the following year in St. Kitts and Nevis (St. Kitts-B), all of which were of the FAW[FL] profile. These observations are more consistent with the St. Kitts-A FAW[M] profile being due to stochastic fluctuations within a small population derived from Puerto Rico rather than an actual mixing of populations from the two continents. The overall conclusion is that fall armyworm movements along the Lesser Antilles can be extrapolated by haplotype comparisons, but the migrations are sufficiently limited that individual island populations can differ substantially in their haplotype profiles from the migratory source population.

### Correspondence of haplotypes profiles with wind patterns

Modest and infrequent migration along the Lesser Antilles is consistent with what would be expected if air transport systems primarily direct long-distance fall armyworm flight. The easterly trade winds, a primary component of which is the Caribbean low-level jet, dominates lower altitude air transport in the Caribbean region. Meanwhile, air transport in Trinidad & Tobago and the northern sections of South America is dominated by the South American low-level jet, which blows to the west and then veers southward upon approaching the Andes mountain range. These wind patterns make flights along the generally north-south orientation of the Lesser Antilles island chain problematic and probably unlikely. Therefore, we anticipate that large-scale population movements between the islands spanned by Puerto Rico and Trinidad & Tobago would have to be limited to times when anomalous wind conditions are more conducive to flight along a north-south orientation. It is possible that such wind conditions are more likely during periods of frequent regional tropical cyclone activity, but these more sporadic events would have to overlap with times of high population density at the migratory source locations. While it is not clear how frequently this might occur, the results of our haplotype survey found only a single instance of the FAW[M] profile in this region, indicating that the coincidence of factors conducive to the significant mixing of the FAW[FL] and FAW[TX] populations probably did not occur during the collection period. Given these considerations, opportunities for genetic exchange between the fall armyworm populations from the two continents could be rare in the Caribbean region.

Describing the magnitude of flying insect migration across the Caribbean region as exemplified by fall armyworm has important ramifications for assessing risks and predicting the spread of both invasive pests and deleterious traits throughout the hemisphere. Fall armyworm itself provides a real world example with the discovery of naturally occurring resistance to the Cry1F *Bt*-toxin in fall armyworm populations in Puerto Rico [31, 47, 48] and subsequent reports of this resistance trait in populations in Brazil [49], Argentina [50, 51], and the United States [52, 53]. The simplest scenario is that the observed resistance stems from a single origin, which then became more widely dispersed by natural migratory behavior. However, our findings that fall armyworm migrations along the Lesser Antilles appears to be limited makes this scenario less likely, as there would only be modest introgressions between the Puerto Rico and South American populations. Perhaps a more parsimonious hypothesis given our data is that the Cry1F resistance trait arose independently in Puerto Rico and one or more of the other locations through independent selection, a possibility that if confirmed would have important ramifications on assumptions about the frequency of *de novo* generation of *Bt*-resistant alleles and resistant management plans [54].

## Conclusions

The rapid dissemination of deleterious traits within a species by natural processes requires consistent and substantial opportunities for genetic interactions between geographically separated populations. These typically occur at the population boundaries where migration or more local dispersion behavior would allow mixing between the groups. The frequency and extent of such potential hybrid zones can be determined if there are genetic markers that can distinguish the two populations. In this study we demonstrate that the mixing of fall armyworm from Florida with those from South America rarely occur in the Caribbean and there is no evidence of substantial introgression of a haplotype that predominates in Florida into populations in South America. These data indicate that the Lesser Antilles is not a major conduit for fall armyworm movements between the two Western Hemisphere continents.

## Supporting information

S1 Table Haplotype data for Brazil and Argentina derived from previous studies and mapped in Fig 1 [6, 17, 28]. (DOCX)

S2 Table Data used for ANOVA analysis in Fig 4. (DOCX)
