# Correction: Fall armyworm migration across the Lesser Antilles and the potential for genetic exchanges between North and South American populations

**DOI:** 10.1371/journal.pone.0175076

**Published:** 2017-03-28

**Authors:** Rodney N. Nagoshi, Shelby Fleischer, Robert L. Meagher, Mirian Hay-Roe, Ayub Khan, M. Gabriela Murúa, Pierre Silvie, Clorinda Vergara, John Westbrook

The following information is missing from the Funding section: This work was partially supported by Biotechnology Risk Assessment Grant Program competitive grant No. 2014-33522-22215 from the USDA National Institute of Food and Agriculture and Agricultural Research Service.
