# Supplementary material for: Fall armyworm migration across the Lesser Antilles and the potential for genetic exchanges between North and South American populations
Source: PLoS One. 2017 Feb 6;12(2):e0171743. doi: 10.1371/journal.pone.0171743 (PMC5293267; doi:10.1371/journal.pone.0171743)
Supplement: S1 Table — (DOCX) [file pone.0171743.s001.docx]

Supplementary Table 1. Haplotype data for Brazil and Argentina derived from previous studies and mapped in Figure 1.

| Country | Province | date | total | ratio | %CSh1 | %CSh2 | %CSh3 | %CSh4 |
| --- | --- | --- | --- | --- | --- | --- | --- | --- |
| Brazil | MG | 2005-7 | 145 | -0.9 | 21 | 73 | 3 | 3 |
| Brazil | Paraná | 2005 | 26 | -0.9 | 23 | 73 | 0 | 4 |
| Argentina | Catamarca | 2011 | 12 | -1.0 | 25 | 75 | 0 | 0 |
| Argentina | Chaco | 2011 | 22 | -1.0 | 0 | 100 | 0 | 0 |
| Argentina | Corrientes | 2011-2 | 43 | -1.0 | 5 | 95 | 0 | 0 |
| Argentina | Salta | 2012 | 9 | -1.0 | 11 | 89 | 0 | 0 |
| Argentina | Santa Fe | 2011-2 | 41 | -1.0 | 37 | 63 | 0 | 0 |
| Argentina | SdE | 2011-2 | 18 | -1.0 | 6 | 94 | 0 | 0 |
| Argentina | Tucuman | 2011-2 | 36 | -1.0 | 11 | 89 | 0 | 0 |

MG, Mato Grosso. SdE, Santiago del Estero
