# Supplementary material for: Fall armyworm migration across the Lesser Antilles and the potential for genetic exchanges between North and South American populations
Source: PLoS One. 2017 Feb 6;12(2):e0171743. doi: 10.1371/journal.pone.0171743 (PMC5293267; doi:10.1371/journal.pone.0171743)
Supplement: S2 Table — (DOCX) [file pone.0171743.s002.docx]

Supplementary Table 2. Data used for ANOVA analysis in Fig. 4.

| State/Country | Date | total | ratio | %CSh1 | %CSh4 |
| --- | --- | --- | --- | --- | --- |
| NA-FAW[TX] |  |  |  |  |  |
| Alabama | 2005-7 | 367 | -0.3 | 11 | 29 |
| Iowa | 2008-9 | 90 | -0.7 | 16 | 13 |
| Illinois | 2008 | 63 | -0.5 | 11 | 21 |
| Indiana | 2008, 2011 | 121 | -0.6 | 13 | 17 |
| Kansas | 2008 | 32 | -0.8 | 19 | 9 |
| Kentucky | 2008 | 29 | -0.5 | 10 | 21 |
| Louisiana | 2007, 2009 | 42 | -0.9 | 5 | 7 |
| Minnesota | 2011 | 27 | -0.5 | 7 | 22 |
| Mississippi | 2004-8 | 267 | -0.4 | 14 | 25 |
| Nebraska | 2011 | 83 | -0.6 | 11 | 17 |
| Oklahoma | 2006 | 68 | -0.7 | 18 | 13 |
| Pennsylvania | 2006-7, 2010 | 304 | -0.6 | 17 | 17 |
| Tennessee | 2008 | 41 | -0.4 | 7 | 27 |
| Texas | 2004-11 | 405 | -0.6 | 12 | 17 |
|  | Mean ± SEM | | -0.6 ± 0.04 | 12 ± 1 | 18 ± 2 |
| NA-FAW[FL] |  |  |  |  |  |
| Florida | 2004-11 | 478 | 0.5 | 6 | 72 |
| Florida | 2012-15 | 800 | 0.4 | 5 | 67 |
| Georgia | 2007 | 258 | 0.3 | 10 | 57 |
| Maryland | 2008 | 21 | 0.2 | 5 | 52 |
| North Carolina | 2004-8 | 157 | 0.2 | 9 | 53 |
| Sourh Carolina | 2011 | 71 | 0.4 | 4 | 69 |
| Virginia | 2008 | 73 | 0.2 | 15 | 49 |
|  | Mean ± SEM | | 0.3 ± 0.1 | 8 ± 2 | 60 ± 3 |
| Car-FAW (collections) |  |  |  |  |  |
| Dominica AB | 2014 | 33 | 0.5 | 0 | 73 |
| St. Kitts A | 2014 | 37 | -0.1 | 0 | 46 |
| St. Kitts B | 2015 | 96 | 0.4 | 0 | 69 |
| Dominican Republic AB | 2015-6 | 62 | 0.4 | 3 | 65 |
| Puerto Rico A | 2007 | 107 | 0.5 | 9 | 63 |
| Puerto Rico B | 2009 | 122 | 0.6 | 2 | 77 |
| Barbados BCDE | 2015 | 231 | 1.0 | 0 | 100 |
|  | Mean ± SEM | | 0.5 ± 0.1 | 2 ± 1 | 72 ± 6 |
| SA-FAW (collections) |  |  |  |  |  |
| Trinidad AB | 2013 | 31 | -0.9 | 16 | 3 |
| Paraguay AB | 2005-6 | 32 | -0.9 | 38 | 3 |
| Bolivia | 2012 | 57 | -1.0 | 14 | 0 |
| Peru | 2014 | 77 | -1.0 | 31 | 0 |
| Brazil | 2005-7 | 237 | -0.9 | 24 | 4 |
| Argentina A | 2010-11 | 101 | -1.0 | 21 | 0 |
| Argentina B | 2012 | 82 | -1.0 | 17 | 0 |
|  | Mean ± SEM | | -1.0 ± 0.02 | 21 ± 3 | 1 ± 1 |
